# Vegetation and the Importance of Insecticide-Treated Target Siting for Control of *Glossina fuscipes fuscipes*

**DOI:** 10.1371/journal.pntd.0001336

**Published:** 2011-09-20

**Authors:** Johan Esterhuizen, Basilio Njiru, Glyn A. Vale, Michael J. Lehane, Stephen J. Torr

**Affiliations:** 1 Vector Group, Liverpool School of Tropical Medicine, Liverpool, United Kingdom; 2 International Centre of Insect Physiology and Ecology (icipe), Thomas Odhiambo Campus, Mbita Point, Kenya; 3 Natural Resource Institute, University of Greenwich, Chatham, Kent, United Kingdom; 4 South African Centre for Epidemiological Modelling and Analysis (SACEMA), University of Stellenbosch, Stellenbosch, South Africa; Universidad de Buenos Aires, Argentina

## Abstract

Control of tsetse flies using insecticide-treated targets is often hampered by vegetation re-growth and encroachment which obscures a target and renders it less effective. Potentially this is of particular concern for the newly developed small targets (0.25 high × 0.5 m wide) which show promise for cost-efficient control of Palpalis group tsetse flies. Consequently the performance of a small target was investigated for *Glossina fuscipes fuscipes* in Kenya, when the target was obscured following the placement of vegetation to simulate various degrees of natural bush encroachment. Catches decreased significantly only when the target was obscured by more than 80%. Even if a small target is underneath a very low overhanging bush (0.5 m above ground), the numbers of *G. f. fuscipes* decreased by only about 30% compared to a target in the open. We show that the efficiency of the small targets, even in small (1 m diameter) clearings, is largely uncompromised by vegetation re-growth because *G. f. fuscipes* readily enter between and under vegetation. The essential characteristic is that there should be some openings between vegetation.

This implies that for this important vector of HAT, and possibly other Palpalis group flies, a smaller initial clearance zone around targets can be made and longer interval between site maintenance visits is possible both of which will result in cost savings for large scale operations. We also investigated and discuss other site features e.g. large solid objects and position in relation to the water's edge in terms of the efficacy of the small targets.

## Author Summary

Sleeping Sickness (Human African Trypanosomiasis) is a serious threat to health and development in sub-Saharan Africa. Due to lack of vaccines and prophylactic drugs, vector control is the only method of disease prevention. Small (0.25×0.5 m) insecticide-treated targets have been shown to be cost-efficient for several Palpalis group tsetse flies, but there are concerns that they may become obscured by vegetation with a subsequent reduction in efficiency. We showed that the efficiency of the small targets was largely uncompromised by vegetation encroachment because *G. f. fuscipes* readily enter between and under vegetation to locate a small target, e.g. into small (1 m diameter) site clearings and underneath a very low (0.5 m) canopy. This implies that the dense vegetation, typical of the riverine habitats of Palpalis group tsetse, will not compromise the performance of tiny targets, as long as there are adequate openings of >30 cm between vegetation. Moreover, the maintanence of cleared areas around targets seems less important for the control of *G. f. fuscipes* with consequent savings in costs for control operations.

## Introduction

The major vectors of Human African Trypanosomiasis (HAT) are in the Palpalis group tsetse flies, especially the *G. fuscipes* subspecies, which are responsible for transmission of >90% of reported HAT cases [1], [2]. In the present situation with limited drug and no vaccine availability, vector control remains an important addition to current efforts against HAT. Tsetse control with insecticide-treated blue/black cloth panels (c. 1–2 m wide ×1 m high), called targets [3], have been used successfully for several Morsitans group tsetse fly species, but only to a limited extent for Palpalis group tsetse [4]. Control of Palpalis group flies is costly and requires high densities of 10–30+ targets to be deployed per km^2^. In contrast, Morsitans group tsetse can be controlled with odour-baited targets at densities as low as 4per km^2^ [5], [6], [7]. It is clear from published studies that factors such as the vegetation, the coverage of the habitat achieved with deployed targets and the correct siting and maintenance of targets play a very important role in efficient control [8]. Targets or traps have to be deployed in sites which allow for the maximum number of tsetse flies available in the range of attraction to locate them. If an odour is used with the device for control of Morsitans group flies, this range is about 5–150 m plus, while an unscented target or trap has a range of about 5–30 m [9]. Limited artificial odours exist at present for Palpalis group flies [10], [11] so the trap or target's efficacy relies heavily on its visibility.

The accepted principle for identifying a suitable site for a trap or target for tsetse species is that the site has open access and visibility in most directions with no large bushes nearby and no low overhanging canopy. For example, optimal sites for the Morsitans group flies *G. m. morsitans* and *G. pallidipes* are open and well away from trees and bushes [8]. For *G. austeni* (also a Morsitans group fly) sites inside the shaded forest, but still ‘open’ due to a high tree canopy and little undergrowth, is best [12]. The optimal trapping sites reported for the Palpalis group fly, *G. f. fuscipes*, are open sites close to the water's edge [13], or an open site outside the forest but not more than 5 m away from the forest edge [14]. Optimal sites for *G. tachinoides* and *G. p. gambiensis* are on the river's edge in direct sunshine [15]. In practice the best available site in the chosen control area, or the next best potential site, will be selected and improved by cutting back vegetation and clearing undergrowth to increase visibility of the target or trap. However, the majority of sites will also include some other features such as large tree trunks, thick bushes, large rocks etc. This immediate arrangement of vegetation and solid objects around the site, i.e. the site morphology, can significantly affect tsetse catches [8]. For example, if a leafy bush with overhanging canopy grows within 1 m of a target catches of *G. m. morsitans* and *G. pallidipes* decreased by 70–80%, while if encroaching vegetation reduced the site clearing to 2 m diameter and covered about 66% of the perimeter catches also decreased by 70% [8].

Despite the importance of the Palpalis group tsetse in disease transmission there is limited information available on the effects of site morphology on target or trap efficiency for these flies, apart from the general description of what is believed to be a good site mentioned above. Understanding the impact of site morphology, especially vegetation encroachment, is imperative following the newly developed cost-efficient small targets (c. 0.125 m^2^) for control of five major HAT vectors namely, *G. fuscipes fuscipes, G. f. quanzensis, G. f. martinii, G. palpalis gambiensis* and *G. tachinoides* [16], [17], [18]. These small targets, as much as 8× smaller than the standard 1×1 m target and using 24× less material than the biconical trap, show great potential for economic savings in control of Palpalis group tsetse. However, the effectiveness of such small targets might be severely and rapidly compromised in the field if vegetation re-growth is as serious a problem as it is with Morsitans group flies as described above. Potentially this factor could rapidly negate the economic savings of using small targets. To address these concerns we have evaluated the performance of small targets for *G. f. fuscipes* in different scenarios of site morphology and vegetation encroachment as may be typically encountered in the tropical environment. The better understanding of the behaviour of *G. f. fuscipes* in relation to site features will contribute to effective and efficient deployment of control and monitoring devices in large scale control of *G. f. fuscipes*.

## Methods

Studies were performed from May to December 2010 on two small islands (each c. 0.5 km^2^), called Big and Small Chamaunga (0° 25′ S, 34°13′ E), off Mbita point in Lake Victoria, Kenya. See [10], [16] for detailed description.

The standard sampling device was a 25×25 cm target made from blue cotton cloth with an adjacent flanking net (25×25 cm) of fine black netting. Henceforth, the term ‘target’ refers to this combination of cloth and netting. Electrocuting grids fitted in a frame covered both the cloth and netting and killed flies on impact, which then fell into trays of water below the grids. See [16] for detailed description. Experiments ran for 12 days each during the peak activity time of *G. f. fuscipes*, from 09:00–12:00 hours. The standard experimental design was a series of Latin-squares of treatments x days x sites, with sites at least 50 m apart. Analysis of variance was performed after transforming the daily catches (*n*) to log (*n*+1). Only detransformed catches are discussed in the text, while the transformed standard errors of the difference (SED) are provided in Tables 1 and 2. The term ‘significant’ denotes that means are different at the P<0.05 level of probability or less.

**Table 1:** Detransformed means of *G. f. fuscipes* catches with different arrangements of vegetation and solid objects around a 0.25×0.5 m Blue+Flanking net target.

		Treatment					
Exp.		Control	A	B	C	SED	P
1	Males	12.3a	19.5a	7.3ab	3.1b	0.136	<0.001
	Females	19.0a	21.1ab	10.2ab	2.6c	0.161	<0.001
	% Obstruction	0	50	50	100		
	Clearing diameter (m)	2.5	2	2	1.5		
2	Males	5.5a	5.5	3.1c		0.076	0.004
	Females	8.3a	5.2a	2.7bc		0.081	<0.001
	% Obstruction	0	60	80			
	Openings width (m)	N/A	1	0.3			
3	Males	3.2	3.5	5.3	4.3	0.105	ns
	Females	4.7	5	3.5	4.8	0.108	ns
	% Obstruction	0	50	50	50		
	Clearing diameter (m)	2.5	2.5	1.8	1		
	Openings width (m)	N/A	1.5	0.75	0.5		
4	Males	1.67a	0.43ab	0.16bc	0bc	0.080	0.002
	Females	3.16a	0.91bc	0.26c	0c	0.080	<0.001
	Obstruction height	0	0.15 m	0.3 m	0.6 m		
	Clearing diameter (m)	2.5	0.75	0.75	0.75		
5	Males	4.6	4.8	4.9	3.5	0.080	ns
	Females	5.1	3.8	3.6	3	0.094	ns
	% Obstruction	0	0	0	0		
	Height of canopy (m)	N/A	2	1	0.5		
6	Males	6.5	9.4	7.6		0.090	ns
	Females	5.3	5.7	6.1		0.074	ns
	% Obstruction	0	25	50			
	Height of canopy (m)	N/A	1	1			
7	Males	1.9	1.9	2.1		0.210	ns
	Females	5.3	6.7	6.1		0.150	ns
	% Obstruction	0	25	25			
8	Males	3.9	3.2	0.8		0.240	ns
	Females	9.9a	2b	0.3c		0.080	<0.001
	% Obstruction	0	50	90			
	Clearing diameter (m)	2.5	2.5	2.5			
9	Males	1.9	1.5	3.3		0.260	ns
	Females	1.1	2.2	2.8		0.160	ns
	Target orientation	No tree	Cloth	Net			
			adjacent	adjacent			

Means not associated with the same letter differ at P<0.05. All experiments ran for 12 days each.

**Table 2:** Detransformed means of *G. f. fuscipes* catches at different distances from the water's edge.

		Treatment	A	B	C	D	SED	P
Exp.	Device	Sex	Control	2 m in water	2 m inland	4 m inland		
1	Target	Male	2.6	6	2.1	2.4	0.246	ns
	Target	Female	4.8	5.8	7.2	6	0.151	ns
2	Trap	Male	6.2	1.3	5.2	3.6	0.217	ns
	Trap	Female	5.7	1.6	2.5	4.8	0.206	ns

Both experiments ran for 12 days each.

**Figure 1:**
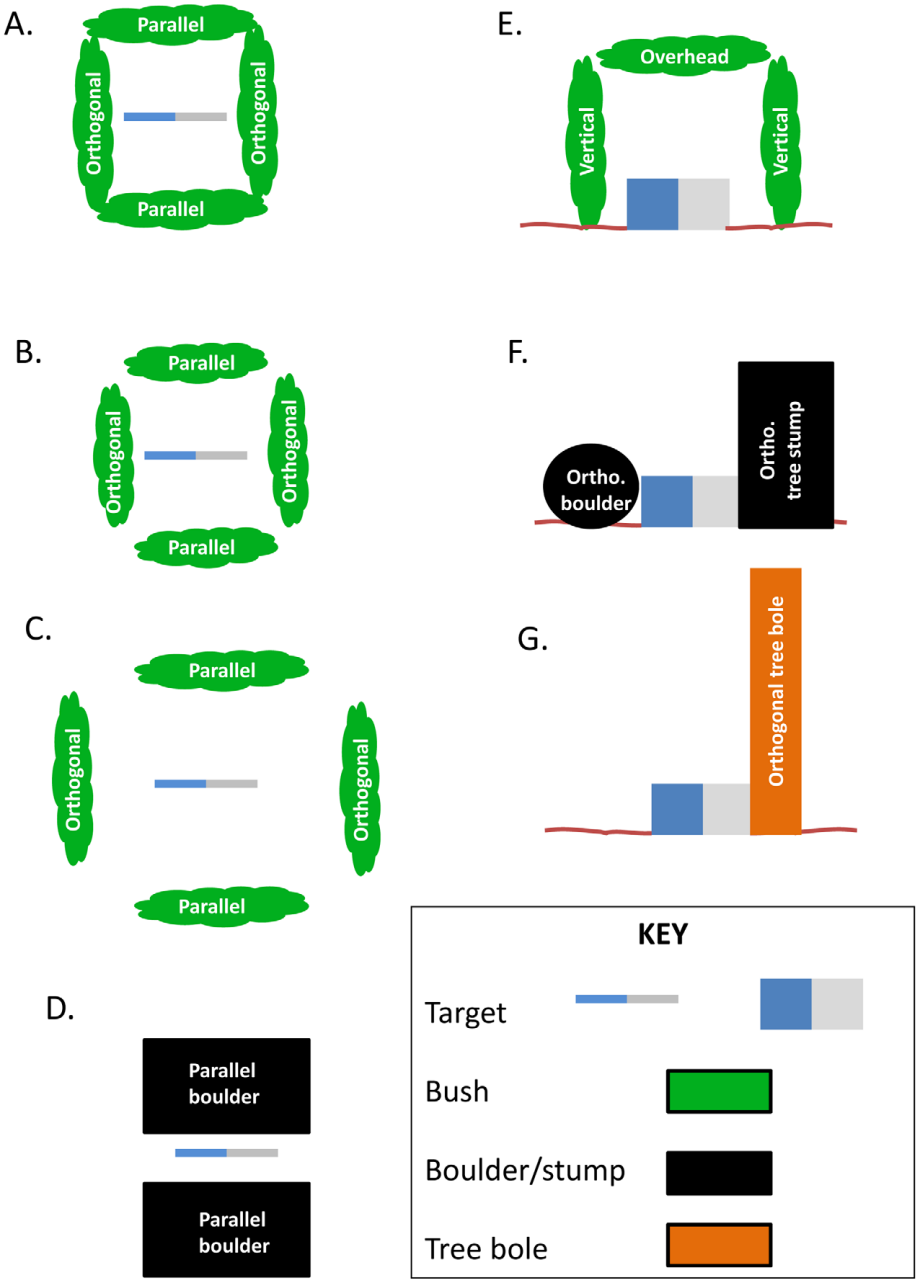
The design of the experiments investigating the effect of obstruction and opening widths between vegetation. A. The target surrounded on four sides by hedges. B. The target surrounded on four sides by hedges with a medium gap between hedges. C. The target surrounded on four sides by hedges with a large gap between hedges. D. The target with an obstruction in front and behind. E. A target with an overhead obstruction. F. The target with obstructions placed either side. G. The target with an obstruction on one side only.

We investigated the following four aspects of site morphology and scenarios for vegetation encroachment; diagrams of the arrangements of targets and surrounding vegetation and other objects are shown in Fig. 1. All treatments were compared to a standard target without any surrounding bushes or other objects in a clearing c. 5 m in diameter.

**Figure 2:**
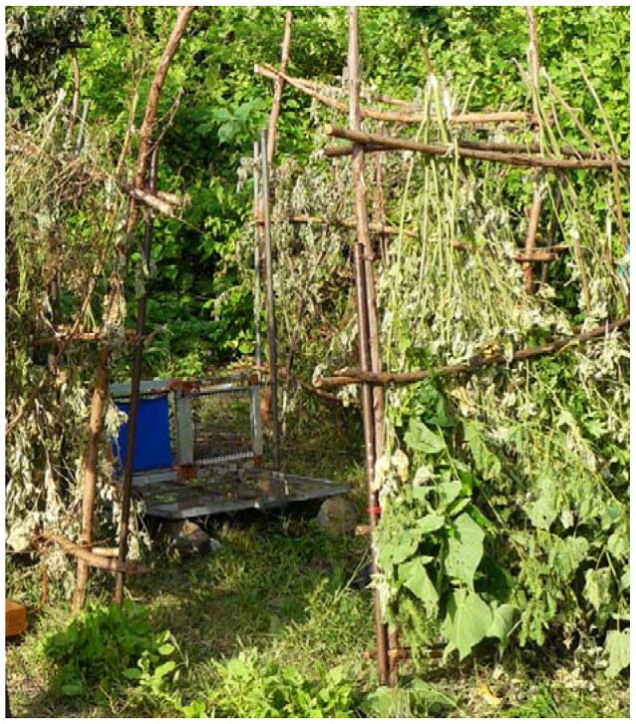
A small target closely surrounded by 15 cm hedges to investigate the effect of thick grass regrowth.

1. *Vegetation encroachment from the sides, for example when a target site is not maintained and vegetation re-growth results in: a) obstruction of the perimeter and b) decreasing the diameter of the site's clearing.* In situations such as these the visibility of the small target and access for tsetse to it and around it, becomes restricted. To simulate bushes, we fixed leafy branches to stick frameworks to form hedges (Fig. 2) which we placed in various arrangements around the target (Figs. 1A–C), as described below. Similar hedges used to simulate site effects for the Morsitans group tsetse *G. m. morsitans* and *G. pallidipes* showed that there was no significant difference in the responses of tsetse to artificial bushes and real ones [8].

The first experiments studied the effect of percentage obstruction of the perimeter of a target site. The target was either completely unobstructed (100% visibility, control treatment) or (A) bushes (1.5 m long, 1 m from the target) were placed on all four sides (0% visibility,) or on two sides (50% visibility) with the hedges being placed either (B) orthogonally or (C) in parallel to the long axis of the target.

**Figure 3:**
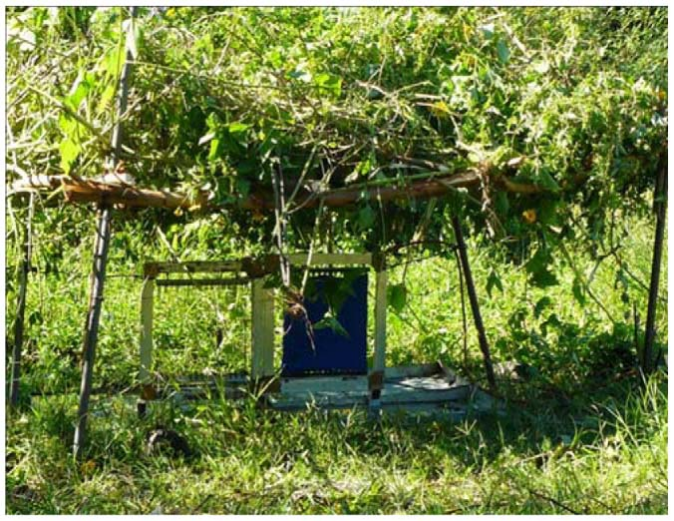
A small target underneath a 0.5 m high leafy canopy to investigate the effect of overhanging vegetation.

The next experiments looked at the effect of surrounding the targets with an incomplete ring of bushes as follows:

- Four hedges were placed 1 m away from the target with four gaps of 0.3 m (i.e., 80% obstruction) or 1 m (33% obstruction) (Fig. 1B).
- This was then followed by an experiment to investigate decreasing clearing sizes. Four hedges were placed 1–2.5 m from the target and the size of the gaps was varied so that the percentage obstruction was maintained at c. 50% (Fig. 1C).
- The impact on tsetse catches of grass re-growth around a small target was also investigated because grass generally re-grows faster than shrubs and bushes and can quickly obscure a small target. Catches from a target in the open were compared to a target surrounded by short hedges (15 cm high), medium hedges (30 cm high) and high hedges (60 cm). All the hedges surrounded each target closely to create a small clearing size of only 0.75 m.

2. *Vegetation encroachment from above; e.g. when a target is deployed under a tree or shrub with overhanging branches*. Metal poles of appropriate length were used to support a framework of green sticks with interwoven leafy branches which formed a canopy above the target (Fig 3). Canopies were 1.5×1.5 m in diameter and 2 m, 1 m or 0.5 m above ground level (Fig. 1E, with overhead vegetation only). A subsequent experiment then investigated a combination of a canopy above a target and a bush next to it, for example when a large bush grew next to as well as over the target. The canopy was 1 m above the target and either (A) one or (B) hedges were placed orthogonally c. 0.75 from the target (Fig 1E).

3. *Proximity to solid objects; e.g. large rocks which may obscure a target, or a thick tree trunk next to the target*. Due to the great variety in size, colours, shapes and combinations of site morphology in nature, it is not possible to duplicate these exhaustively or change these features between sites. A partial simulation of large rocks could be achieved by placing drums horizontally on the ground, or vertically on top of each other to simulate these large objects (Fig 1, diagrams D&F). The drums were made of plastic (50 cm diameter × 80 cm high, volume  = 160 L) covered with matt black cotton cloth and placed either next to, or in front of a target.

In addition, we also looked at the responses of *G. f. fuscipes* to a small target next to a real tree bole (a paw-paw tree bole 30 cm diameter, 1.8 m high) and whether the orientation of the target to the tree was of importance, i.e. with the blue cloth or the black netting panel closest to the bole (Fig 1G).

4. C*atches of G. f. fuscipes at different distances from the water*'*s edge.* This was done because standard field procedure is to place the device close to the water's edge [19], [20] partly to increase visibility, but also because casual field observations show flies apparently move along the water's edge. A standard small target was deployed in a randomized block design in four sites. The control site was the water's edge, with the other three sites at 2 m or 4 m inland or 2 m into the water. For the latter, the target and collection tray were fixed to a floating platform of sticks and the electric cables lengthened to reach the power supply on the shore. The same set-up was repeated but using a standard Biconical trap as collection device.

## Results

Table 1 lists all the experiments on site morphology and their results.

### Vegetation encroachment from the sides and above

#### Effect of surrounding vegetation

Partial obstruction of a target by bushes placed in parallel or orthogonally to the target, so that the side of the target was unobscured (Table 1, experiment 1, treatment A), did not reduce catches of female *G. f. fuscipes* (21.1 tsetse/day) compared to the control (unobscured) target 19.0 tsetse/day; no significant difference between means. In contrast, if the bush was placed parallel to the target, thereby obstructing the side view, catches decreased by 47% for females and 41% for males (Treatment B, 10.2 tsetse/day, P<0.001). Complete obstruction of a target by leafy vegetation (Treatment C) resulted in a decrease of 87% in catches of females (2.6 flies/days, P<0.001) and 76% less males, compared to the target in an open site. This reduction is of course expected due to the decreased visibility of the target and physical obstruction of the hedges. However, even with this degree of visual obstruction some flies were still killed by the target.

Vegetation may encroach upon a target from all four sides, resulting in a decrease in the clearing diameter and in the width of the openings between the vegetation surrounding the target. Our data shows that a decrease in clearing diameter down to 1 m, the minimum we looked at, had no significant effect on catches of male or female *G. f. fuscipes,* but that the width of openings between bushes is important. For example, female catches in a 1 m diameter clearing with 50 cm wide openings between the four hedges surrounding it (Table 1, experiment 3, treatment C, 4.8 tsetse/day), were similar to that of the 1.8 m clearing with 75 cm wide openings between the four bushes (Treatment B, 3.5 tsetse/day), as well as to catches in the 2.5 m wide clearing with openings of 1.5 m between the bushes (Treatment A, 5.0 tsetse/day) and as the control site (4.7 tsetse/day). However, if the openings between the four bushes were only 30 cm wide, a significant decrease in catches of 72% for females (Table 1, experiment 2, treatment B, 2.7 tsetse/day, P<0.001) and 54% reduction for males (3.1 tsetse/day, P<0.004) was evident.

Simulations of regrowth of grass closely around a target resulted in significant decrease in tsetse catches (Table 1, exp.4). Fly numbers available during this experiment were the lowest during the whole study period, making interpretation difficult. The results indicate that grass of 0.15 m high (Treatment B, 0.91 tsetse/day, s.e.d. = 0.08) caught significantly less females than the control target (3.6 tsetse/day). The target behind the 0.3 m hedge also caught significantly less females (Treatment C, 0.26 tsetse/day), while the target behind the 0.6 m hedges caught no flies.

#### Effect of an overhead canopy

A canopy of leafy vegetation above a small target did not affect catches significantly compared to catches in an open site. Data show (Table 1, experiment 5) that *G. f. fuscipes* enter under overhanging vegetation, even as low as 0.5 m to reach a small target. Remarkably, this 0.5 m low canopy treatment caught only 36% less females (Treatment C, 3.0 tsetse/day, s.e.d. = 0.09) and 26% less males (3.5 tsetse/day, s.e.d. = 0.08) than the open target. The 1 m and 2 m high canopies caught equal numbers of male flies as the open target and 25–29% fewer females (Treatment A, B). The combination of leafy vegetation partially obscuring a target from the side, with a leafy canopy 1 m above ground level (Table 1, experiment 6, treatment B), did not significantly decrease catches of males (7.6 tsetse/day, s.e.d. = 0.09, P = 0.18) or females (6.1 flies/day, s.e.d. = 0.07, P = 0.6). Similarly, with two bushes (Treatment A) next to the target and an overhanging canopy, no significant decrease in catches was evident.

All the above data indicates that *G. f. fuscipes* readily enters between bushes and vegetation and that the small blue+net target (0.25×0.5 m) remains as effective as a target in an open site, even if partially obscured (up to 70%) from the sides and above by vegetation. The important point is that some openings between vegetation should remain.

### Proximity to solid objects

Following the vegetation encroachment experiments, we looked at the effect on catches of large solid objects next to a small target. As described in Material and Methods, we used drums as artificial rocks and tree trunks for this study, due to the difficulty in otherwise simulating these objects in the field. Our data showed that when either the ‘rock’ or ‘tree trunk’ was placed next to the target there was no significant difference compared to catches from the control target (Table 1, experiment 7). In fact, the catches of female *G. f. fuscipes* increased in both cases, by 1.2× when the rock (Treatment A) was used (6.7 tsetse/day, s.e.d. = 0.1) and by 1.1× when the tree was used (Treatment B, 6.1 tsetse/day). Tsetse flies are attracted to large black objects and black drums and flat black cloth panels are used routinely in experiments to increase visual attraction [8], [16]. Therefore the observed increase in catches may be expected, but the more interesting question is what happens when such large black objects obscure the visibility of the small target, e.g. when a large rock is directly obscuring a small target. We found (Experiment 8) that the unobscured target (9.9 flies/day, s.e.d. = 0.08) caught 80% (P<0.001) more females than the target with one drum in front (Treatment A, 2.0 flies/day) and 98% more females than with a drum on each side (Treatment B, 0.03 flies/day). Catches of male *G. f. fuscipes* showed no significant difference (P = 0.2) between the target in the open and either of the treatments, although 20% less flies were caught with one drum in front of the target (Treatment A, 3.2 flies/day) and 80% less with a drum on each side of the target (Treatment B, 0.2 flies/day), completely obscuring the frontal views. When placing a target next a real tree trunk (Table 1, exp. 9) there was a doubling in female catches with both the blue cloth closest to the trunk (Treatment B, 2.2 flies/day) and with the netting closest to trunk (Treatment C, 2.8 flies/day) although this was not significant (P = 0.26).

### Water's edge

Finally, we investigated the effect of the position of a small target and biconical trap in relation to the water's edge (Table 2, experiment 1 & 2). For *G. f. fuscipes,* the trapping sites usually used in control campaigns are open and close to, or right on the water's edge [13]. Casual field observations indicate that flies may use the water's edge as a movement ‘corridor’, perhaps due to more abundant green vegetation for shelter, higher humidity and higher chance of finding a host, particularly monitor lizards which inhabit these aquatic margins. However, our results show that a small target placed on the water's edge did not catch significantly more female flies than targets placed 2 m (7.2 flies/day, s.e.d. = 0.15), 4 m inland (6.0 flies/day), or floating 2 m into the water (5.8 flies/day). When a biconical trap was used as collection device (Table 2, experiment 2), female catches on the water's edge were slightly better (5.7 flies/day, s.e.d. = 0.2) than that at 2 m inland (2.5 flies/day), 4 m inland (4.8 flies/day) and 2 m in the water (1.6 flies/day). However, the differences were not significant. Although deployment at the water's edge may be desirable, it appears not to be essential because target efficiency does not decrease significantly over a few meters at least. This is important as it means targets can be sited to minimise losses due to flooding.

## Discussion

Vegetation encroachment around a small target, from the sides and above, does not significantly affect its killing efficiency for *G. f. fuscipes* as long as there are some openings between adjacent bushes, wider than 30 cm. These results are intriguing because the rapid re-growth potential of the tropical vegetation in the habitat of *G. f. fuscipes* and other Palpalis group tsetse, combined with the small size of the targets, could make it seem improbable that these targets will remain effective. Indeed, our results show that only one such scenario, grass regrowth very close to the target, poses a serious threat to their performance. Our simulation of grass height corresponds roughly to between c. 15 (15 cm high) and 60 days (60 cm high) as observed in the rainy season in the field. As expected, the small diameter clearings (0.75 m) created by the proximity of surrounding grass significantly decreased target catches. However, this represents severe and complete grass regrowth around a target, something which does not happen frequently in nature because grass rarely grows uniformly and there always remain some openings between clumps of grass to allow visibility and access to a target. In addition and as matter of routine, this scenario is easily prevented by the proper initial clearing of target sites. In some circumstances this can aided by the subsequent use of systemic herbicides such as glyphosate which can inhibit grass regrowth for several months afterwards. For example, application of glyphosate maintained reduced grass cover for up to 26 weeks on a rainforest edge [21]. Limited studies have been done on the effect of vegetation encroachment on the efficiency of a target or trap for Palpalis group tsetse species. The most relevant studies are from Morsitans group flies [8] where the effect of vegetation close to a trap dramatically and significantly reduced catches of *G.m. morsitans* and *G. pallidipes*. For example, one bush with an overhanging canopy next to a trap, decreased catches of both Morsitans group species by more than 80%, while a decrease in diameter of clearing size from 12 m to 2 m led to about 65% decrease in catches. In contrast, our data for *G. f. fuscipes* showed no significant difference between control and treatment catches in both scenarios, even with only 1 m diameter clearings.

The presence of a few bushes surrounding a target site, not obscuring more than about 70% visibility, may in fact be slightly beneficial. An apparently similar situation was evident with *G. m. morsitans* and *G. pallidipes*, where trap catches increased if 2–6 bushes were within 2–12 m from the target [8]. However, the smallest clearing size used for the *G. m. morsitans* and *G. pallidipes* experiments was 2 m radius (4 m diameter), at which catches of both species were 65% less than the open trap. As the clearing diameter was increased to 12 m, the catches increased. For *G. f. fuscipes* a remarkably small clearing of even 1 m diameter remained effective. The importance of an opening about 50 cm wide between adjacent bushes around a target was evident as catches reduced significantly (by 68% for females) if this opening was 30 cm and less. This was also found for *G. m. morsitans* and *G. pallidipes*, with catches of both species increasing significantly when the opening size is widened from 25 cm to 50 cm and more [8].

We showed that *G. f. fuscipes* readily enters between and through leafy vegetation to locate a small target. This behaviour corresponds with the habitat along the islands and shore of Lake Victoria, where their main hosts are monitor lizards. *G. f. fuscipes* have to locate these medium to small-sized reptiles between the leafy vegetation and rocks. Other site features such as large rocks or tree boles close to the target also affect catches of *G. f. fuscipes*, e.g. a single large solid object to the side of a small target, whether this was an artificial rock or tree bole, or a natural tree bole, actually increased catches. On the other hand, if one or more such objects obscured the frontal view of the target, catches decrease significantly. In addition, it would seem that the waters edge is not a required trap or target site for *G. f. fuscipes.* This is important as changes in water height can easily sweep away control devices with much cost to control programmes. The priority should be given to visibility rather than proximity to waters edge (at least within the 4 m investigated here), because target efficiency does not decrease significantly over just a few meters between the water's edge and inland.

As illustrated in this work, the small targets retain their killing efficacy in several situations of vegetation encroachment, even in small clearings of 1 m diameter and with leafy bushes close-by and above. Nevertheless, in practice, we recommend that sites be cleared to at least 2–3 m in diameter during initial deployment and that overhanging or intruding vegetation be cut back. This will allow for maximum visibility of the target during the first months after deployment. Maintenance intervals will vary between locations depending on vegetation regrowth rates, but under conditions in the study area we expect the small targets to remain efficient for 3–6 months after initial deployment, with no maintenance visits required in-between. If possible the use of a systemic herbicide applied on the site will prevent the regrowth of grass and other vegetation. The possible herbicides available for use next to watercourses are very limited; for example glyphosate is the only product registered for such use in the U.K.

The data presented here demonstrates the potential for less frequent maintenance visits to cut back and control vegetation, which is a major financial constraint in tsetse control operations [22] where targets have to be serviced regularly to maintain efficiency. Another reason for maintenance visits is to ensure that the target is still in its correct position, is upright, the cloth is in good condition and that the moving parts are free. When using large targets, this maintenance has to be carried out regularly and irrespective of whether the vegetation needs clearing. This will be largely unnecessary when using the small targets because they will be more stable and not blown over or bent by strong winds as frequently as large targets.

Clearly, there is potential for low-cost, low-maintenance control of *G. f. fuscipes*, and there is a necessity of these types of studies on other Palpalis group tsetse species in other tropical environments, to allow for better understanding and control of these major vectors of HAT.
